# Six Hundred and Sixty Nanometer Light Exposure‐Induced Alterations in Actin Filament, Mitochondrial Morphological Dynamics, and Migration in Mesenchymal Stem Cells

**DOI:** 10.1002/jbio.202400544

**Published:** 2025-09-05

**Authors:** Mahima Rastogi, Khageswar Sahu, Shovan Kumar Majumder

**Affiliations:** ^1^ Laser Biomedical Applications Division Raja Ramanna Centre for Advanced Technology Indore Madhya Pradesh India; ^2^ Homi Bhabha National Institute, Training School Complex, Anushakti Nagar Mumbai Maharashtra India

**Keywords:** actin filament, adipose‐derived mesenchymal stem cell, photobiomodulation

## Abstract

Actin cytoskeleton alteration and cell homing/migration are crucial determinants for the success of stem cell (SC) based therapy. Photobiomodulation (PBM) is a promising non‐pharmacological approach for modulating SC potency. Though ~660 nm is the most studied wavelength for the proliferation/differentiation of SCs, the migration and cytoskeleton remodeling aspects have not been investigated in detail. In this study, we report the effect of ~660 nm on actin filaments, mitochondrial morphological dynamics, along with the migration of human adipose‐derived mesenchymal stem cells (hADMSCs). Exposure to ~660 nm (~15 J/cm^2^) elicits rapid actin fiber rearrangement leading to elongated, parallel fibers, and mitochondrial granulation along the leading edge of cell migration. In addition, 660 nm (~15 J/cm^2^) also enhances cell proliferation, ATP, and ROS levels. These ultrastructural and biochemical alterations, in conjunction with the increased cell migration, shed new light on mechanistic perspectives to elicit enhanced homing/migration in SCs and would help in further optimization of ~660 nm based SC priming.

## Graphical abstract

Six hundred and sixty nanometer light‐induced alteration of actin filament cytoskeleton, mitochondrial morphological dynamics, and enhanced migration in adipose‐derived mesenchymal stem cells.

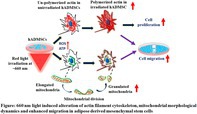

## 1 Introduction

Mesenchymal stem cells (MSCs) possess the ability to differentiate into a diverse range of cell lineages, such as osteogenic, chondrogenic, adipogenic, and neural cells. MSCs hold immense potential in a myriad of medical fields that include regenerative medicine, tissue engineering, and the treatment of numerous chronic and acute diseases [1]. Among the MSCs, human adipose‐derived mesenchymal stem cells (hADMSCs) are preferable for cell therapy due to their ease of isolation, low immunogenicity, and other procedural benefits [2]. However, it is challenging to maintain cell potency and achieve therapeutically relevant cell numbers in in vitro batch culture.

Cell therapy involves the implantation of the MSCs, which aid in wound repair and tissue regeneration by migrating to the site of action, followed by rapid proliferation and differentiation into different cell types such as fibroblasts and endothelial cells [3]. Therefore, devising strategies to improve MSC homing is of tremendous current interest. Normally, the MSCs, postimplantation can leave their niche and migrate to the site of action through endothelial capillaries. However, postimplantation, the MSCs encounter inhospitable conditions in vivo, such as inflammatory and oxidant‐rich environments, that can hamper the potential of these stem cells (SCs) to migrate and proliferate [4]. In addition, it has been observed that with the increasing passage, ‐migration and proliferation potential of SCs decrease considerbaly [5]. Therefore, to fully endow the SCs with enhanced proliferation, differentiation, and migration properties, priming of SCs with different chemical approaches is undertaken. While the chemical stimulants and growth factors succeed in stimulating SCs in vitro, the procedures are very cost‐intensive, and applying the same approaches to implanted cells to achieve the desired spatiotemporal control in vivo without eliciting adverse effects on host cells is a challenging task [6].

In the context of stimulating SCs, non‐pharmacological approaches are attractive prospects, among which photobiomodulation (PBM) based pre‐priming can be an attractive new avenue for enhancing migration of SCs in vitro and in vivo. PBM‐based SC pre‐priming offers several advantages that include: (i) no possibility of side effects from chemical contaminants, (ii) no requirement of downstream ultra‐purification steps to get rid of the trace chemical contaminants in SCs or their secretome, (iii) nonintrusive ways of cell stimulation in vitro and in vivo, and (iv) the ability to achieve high spatial and temporal control. Considering the optical approaches, PBM, based on the application of low‐intensity visible or near‐infrared (NIR) light irradiation from lasers or light‐emitting diodes (LEDs), offers a non‐pharmacological and nongenetic approach to promote SC proliferation, differentiation, and secretome production. PBM is fast gaining traction in SC‐based applications, which are, apart from the above‐mentioned reasons, due to the ease of clinical translatability and cost‐effectiveness. Mechnistically, PBM action is associated with photo‐physical and photochemical effects at the molecular and cellular levels. Despite significant focus on the photochemical mechanisms underlying PBM, the complex mechanisms are yet to be fully elucidated [7]. For instance, the most accepted mechanism of action for red and NIR light‐based PBM is the absorption of photons by cytochrome c oxidase (CCO), a component of the respiratory chain in the mitochondria. Excitation of CCO in mitochondria leads to modulation of ATP synthesis, release of signaling molecules like reactive oxygen species (ROS), which further results in cell proliferation and wound healing. Although light absorption by CCO is well established, recently there have been references to other possible mechanisms other than cytochrome absorption [8]. Therefore, the understanding of the mechanism of action of PBM is far from complete.

Cell migration, mediated by cytoskeleton remodeling, is an important event in wound repair. The cytoskeleton maintains cell structure, transport of organelles to desired cytoplasmic locations, and so forth [9]. Actin filaments represent one type of cytoskeleton and play a defined role in cell migration, division, and wound healing. Furthermore, cytoplasmic actin can regulate cell morphology, movement, and organelle dynamics [10]. In particular, the regulation of mitochondrial function can be governed via myosin in association with actin [11]. Fundamentally, the actin cytoskeleton and actin‐binding proteins play multifaceted roles in mitochondrial trafficking, dynamics, biogenesis, and so on. Xie et al. also demonstrated that β‐actin is essential for mitochondrial quality control [12]. In addition, mitochondrial dynamics of fission and fusion are reported to be governed via the cytoskeleton interaction with mitochondria [13].

Actin polymerization and depolymerization can be altered by the oxidation of actin or actin‐binding proteins [14]. Consequently, oxidants like hydrogen peroxide can induce actin polymerization [15, 16]. However, it is challenging to achieve spatiotemporal regulation of the actin polymerization and depolymerization events in tissues or within cells via chemical oxidants. On the other hand, PBM can be used to regulate the endogenous redox status of the irradiated area with more spatiotemporal precision, which allows even more sophisticated control. Therefore, optical approaches are emerging as futuristic concepts for the optical manipulation of actin function in therapeutic applications [17].

PBM elicits the generation of ROS, ATP, and calcium ions, all of which play defining roles in actin dynamics. Hence, it is reasonable to hypothesize that PBM would modulate cytoskeletal organization [17], culminating in beneficial responses such as increased cell proliferation and migration [18, 19]. A previous report demonstrated that exposure to red and NIR light stimulates proliferation, while blue and green light exposure inhibits proliferation [20]. Interestingly, Zare et al. reported enhanced in vitro cell viability of hASCs and hBMSCs with the combined 630 and 830 nm laser treatment [21]. Furthermore, two studies have demonstrated the effect of green and NIR light exposure on SC cytoskeleton. However, the specific photoreceptors and mechanisms for both wavelengths have yet to be fully characterized [22, 23]. At the same time, the effect of ~660 nm on the cytoskeleton of non‐stem cells has also been reported in two previous studies [18, 24]. It is established that PBM‐induced cell modulation can vary greatly according to the SC type, which has a different propensity for migration and differentiation. However, to the best of our knowledge, the effect of ~660 nm light on actin organization, mitochondrial arrangement, and migration of SCs, in particular hADMSCs, has not been investigated in detail.

Inside a cell, the mitochondria can exist as two interconverting forms: long tubules and small round vesicles, as mitochondria continuously undergo the opposing processes of both fusion and fission [25]. The relative contribution of fusion and fission determines the size of mitochondria. Various stimuli, including cellular redox state, can shift this equilibrium. Hence, in this study, we determine the effect of ~660 nm light exposure on actin cytoskeleton and mitochondrial morphological changes in conjunction with cellular metabolic state and cell migration. Furthermore, in our study, the effect of red light exposure on ROS and ATP levels was studied to impart a biochemical basis to the PBM‐induced modulation of SC migration and cytoskeletal reorganization. The hADMSC has been chosen as this cell makes a good candidate for SC‐based cell therapy and there is no detailed study on the role of ~660 nm exposure on hADMSC migration and cytoskeletal reorganization.

## 2 Materials and Methods

### 2.1 Cell Culture and Experimental Design

hADMSCs (CL007) procured from HiMedia were cultured in MSC expansion medium w/l‐glutamine and sodium bicarbonate (AL512) supplemented with 10% FBS and 1% gentamicin–amphotericin B solution at 37°C in a 5% CO_2_–95% air humidified atmosphere. The culture medium was replaced every 48 h, and the cells were trypsinized upon reaching ~80% confluence. For this experiment, cells with passage numbers 3–6 were used.

### 2.2 PBM Treatment

**FIGURE 1:**
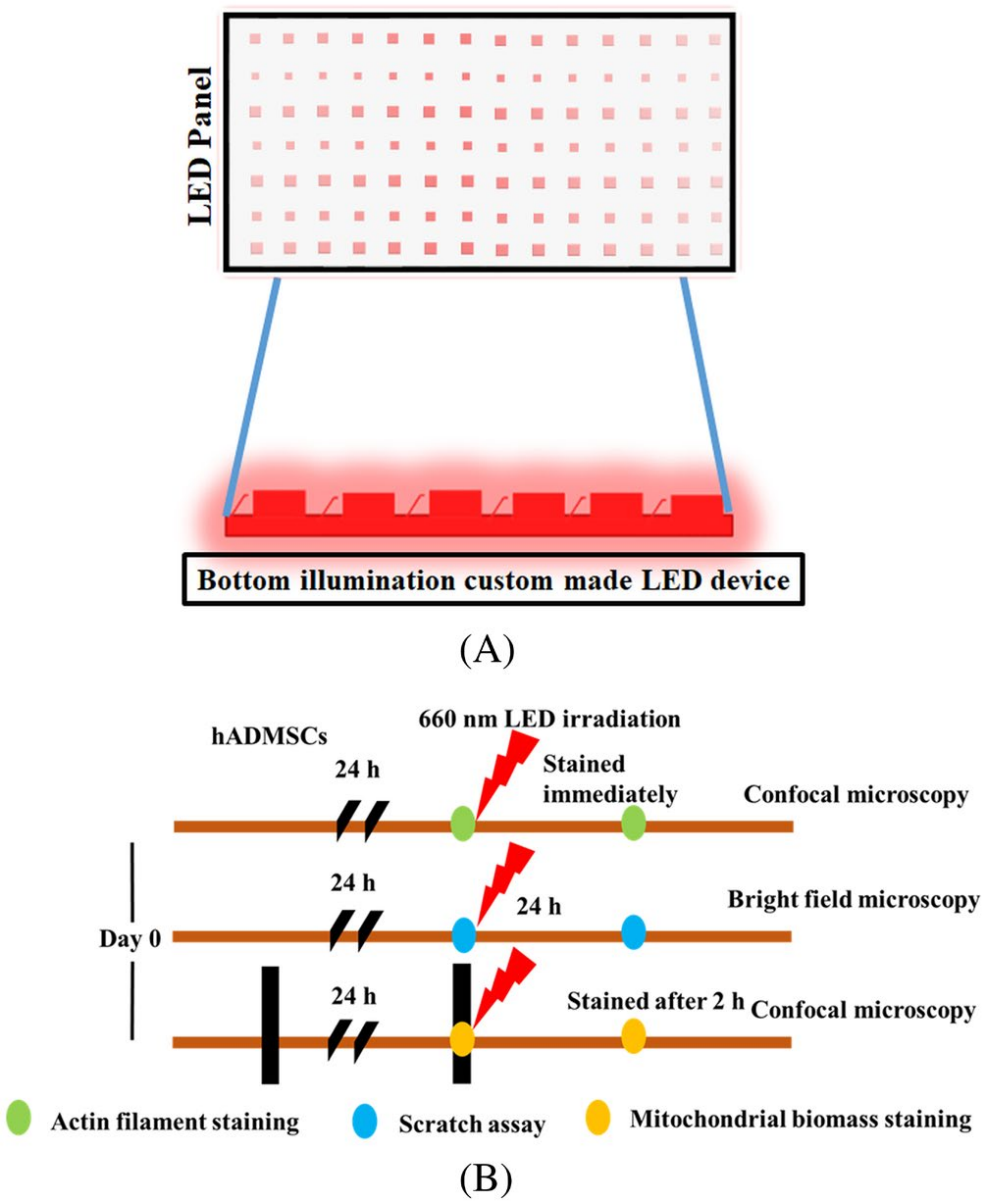
(A) Bottom illumination LED setup. (B) Scheme of the experiments.

PBM treatments were carried out by using an LED array‐based bottom illumination device (Figure 1A,B). Different light fluence (~3, ~6, ~9, ~15, and ~60 J/cm^2^) were achieved by irradiating the cells with power density (~30 mW/cm^2^) for varying exposure times ranging from 100 to 2000 s (Table 1). Incident light power was measured using a power meter. The illumina experiments in vitro were conducted in 96, 24, and 12‐well plates as per the experimental requirement. In studies requiring confocal imaging, cells were grown in a glass‐bottomed confocal imaging dish. To minimize the unwanted light reflection to the side wells, the wells surrounding the sample well to be illuminated were blocked with black paper and aluminium foil, which were affixed at the bottom of the plate.

**TABLE 1:** Light exposure parameters.

Wavelength	655 nm
FWHM	25 nm
Light source type	LEDs
Light source arrangement	LED array, positioned to each well of the sample plate
Power at sample plain	~18 mW
Power density	~30 mW/cm^2^
Time of exposure	100, 200, 300, and 500 s
Fluence	~3, ~6, ~9, ~15, and 60 J/cm^2^
Light exposure frequency	Once at 24 h, if not stated otherwise

### 2.3 Fluorescence Microscopy for ROS Detection

The amount of ROS generated in cells was measured by fluorescence microscopy using the fluorescence probe DCFH‐DA. The nonfluorescent DCFH‐DA freely diffuses into the cells and is converted into a nonfluorescent product, 2′,7′‐dichlorodihydrofluorescein (DCFH) by the enzymatic action of cellular esterases. In cells, DCFH reacts with a broad range of ROS (O_2_•^−^, •OH, H_2_O_2_, and ^1^O_2_) and is oxidized into the highly fluorescent product dichlorofluorescein (DCF). Briefly, the procedure was as follows. The cells were subjected to various treatments and, after the specified time periods mentioned in specific studies, were washed twice with ice‐cold PBS and incubated in culture media (without serum) containing DCFH‐DA (10 μM) for 30 min at 37°C in the dark. At least three images per treatment group were captured. The corrected total fluorescence (CTCF) was calculated using the ImageJ software, applying the following formula:

$$\begin{array}{c} \text{Corrected total cell fluorescence}\;\left(\text{CTCF}\right)=\text{Integrated density of selected cell}\\ -\left[\left(\text{Area of selected cell}\right)\times \left(\text{Mean gray value of background}\right)\right] \end{array}$$

The averaged fluorescence values were plotted using GraphPad Prism 7 software (La Jolla, CA, USA).

### 2.4 Confocal Microscopic Imaging for Actin Filament Organization

The hADMSCs were seeded onto glass coverslips in a confocal dish at a density of ~3000 cells/cm^2^ and incubated for 24 h (37°C, 5% CO_2_). After fixation in 3.7% paraformaldehyde (20 min, 25°C), the cells were permeabilized (0.1% Triton X‐100 in PBS, 15 min), blocked with 1% bovine serum albumin (Sigma) for 40 min (25°C). Cells were stained with rhodamine phalloidin (Thermo Fisher) for 1 h (37°C, dark), followed by incubation with Hoechst 33342 (Thermo Fisher) for 10 min (37°C) and washing. The stained cell samples were imaged under a confocal microscope (LSM 900 Zeiss, Germany) by exciting the stained cells at 360 and 540 nm, meant for Hoechst dye (nucleus staining) and phalloidin (actin staining), respectively. Images were captured at 40× magnification at five different locations on a glass coverslip. For the unirradiated control group, hADMSCs were seeded and incubated using the same experimental conditions but without ~660 nm irradiation. Regions of interest (ROIs) were defined in the raw or filtered images, and the average fluorescence intensity was calculated for each ROI at each time point with the following formula: Corrected total cell fluorescence (CTCF) = Integrated density of selected cell − [(Area of selected cell) × (Mean gray value of background)] [26]. The CTCF formula determines the total cell fluorescence intensity by subtracting the background fluorescence and correcting for intensity differences due to area. The averaged fluorescence values were plotted using GraphPad Prism 7 software.

### 2.5 Confocal Microscopic Imaging of Mitochondrial Morphology

hADMSCs seeded on glass coverslips at a density of ~3000 cells/cm^2^ were incubated for 24 h (37°C, 5% CO_2_). The hADMSCs were exposed to ~660 nm and cultivated for 2 h, followed by staining with MitoTracker green dye (Thermo Fisher), according to the protocol described in [27]. Briefly, cells were washed twice with phosphate‐buffered saline (PBS), stained with MitoTracker green dye (200 nM) in the dark for 30 min (37°C, 5% CO_2_) and then with Hoechst 33342 as described in Section 2.4. Samples were observed using confocal microscopy (LSM 900 Zeiss, Germany) at wavelengths of 360 and 490 nm for excitation of Hoechst and MitoTracker green, respectively (Figure 1B). Images were captured at 40× magnification. For scratch wounds and mitochondrial morphometric analysis, ImageJ (NIH) was used. To measure mitochondria length, five cells were selected from each image, and the length of 8–10 randomly chosen mitochondria/cell was measured.

### 2.6 Scratch Wound Assay in Monolayer Culture of hADMSCs

Cells were cultured to appropriate confluence (~50 000 cells/well) in 12‐well plates (37°C, 5% CO_2_) for 24 h, after which, in each well, a scratch was made using a 100 μL sterile micropipette tip. Cells were then either exposed to 660 nm, according to the parameters indicated in Table 1. The scratches were examined and imaged under a microscope (4× magnification) immediately after irradiation at 0 and 24 h. Wound healing rate was measured using ImageJ software. Experiments were performed in triplicate.

The percentage of wound closure in each group can be quantified by tracking cell migration and applying the following formula:

$$\begin{array}{cc} \text{Wound closure}\;\left(\%\right)=\; & [\left(\text{Area of wound}\;\mathrm{at}\;0\;h-\text{Area of wound}\;\mathrm{at}\;24\;h\right)\\ & /\left(\text{Area of wound}\;\mathrm{at}\;0\;h\right)]\times 100 \end{array}$$

### 2.7 WST Assay on Cell Viability of hADMSCs

Cell viability was evaluated by WST‐8 (2‐(2‐methoxy‐4‐nitrophenyl)‐3‐(4‐nitrophenyl)‐5‐(2,4‐disulfophenyl)‐2*H* tetrazolium, monosodium salt) assay. The cells were seeded in 96‐well plates and cultured in their specific growth medium for 24 h before being subjected to the different PBM treatments. After irradiation, cells were incubated for 24 h and then 10 μL of WST‐8 solution was added to each well of the 96‐well plate. A Multi‐Mode Microplate Reader (Synergy H1, Biotek) was used for measurement. Absorbance was measured at 450 nm.

### 2.8 Carboxyfluorescein Succinimidyl Ester Cell Proliferation Assay

Cell proliferation was evaluated by carboxyfluorescein succinimidyl ester (CFSE) dye dilution assay. hADMSCs were cultured in 24‐well plates for 24 h before being subjected to different PBM treatments. Before treatment, cells were stained with 5 μM CFDA‐SE (Invitrogen). Then, CFSE‐stained cells were subjected to PBM treatment and cultured for another 72 h. After 72 h, fluorescence intensity of CFSE‐stained cells was measured using a Multi‐Mode Microplate Reader (Synergy H1, Biotek). Reduction in fluorescence intensity as compared to the initial intensity of CFSE indicates cell proliferation was plotted.

### 2.9 ATP Measurement

ATP levels were measured using an ATP/ADP ratio kit (Sigma‐Aldrich) as per manufacturer's protocol. Briefly, cells were cultured in a 96‐well plate and subjected to different PBM treatments, and ATP levels were measured immediately and 4 h posttreatment using a Multi‐Mode Microplate Reader (Synergy H1, Biotek). Luminescence levels in different sample groups were plotted.

### 2.10 Statistical Analysis

All the experiments were repeated thrice. Data are represented as mean and standard deviation. Statistical analyses were performed using Prism v9.0 software (GraphPad). Student's *t*‐test was used to compute the statistical significance in the case of the WST‐8 assay, *p < 0.05 (*t*‐test). One‐way analysis of variance (ANOVA) was performed for multiple comparisons in the case of F‐actin, MitoTracker green fluorescence intensity, mitochondrial length, CFSE cell proliferation, ATP measurement *p < 0.05, ***p < 0.001, ****p < 0.0001 (one‐way ANOVA). Image analysis was performed using ImageJ software (National Institute of Health, Bethesda, MD, USA).

## 3 Results and Discussion

Finding innovative strategies for improving the migration ability of MSCs, including the hADMSCs, has become a major focus in regenerative medicine. The homing capability of hADMScs following implantation can exert a significant impact on tissue regeneration and therapeutic applications. Cytoskeleton reorganization is central to cell migration, while the cytoskeleton is very much responsive to external stimuli like infection, inflammation, radiation, and internal stimuli such as ROS [17]. One of the mechanisms by which PBM is expected to act is via ROS generation, which in turn, can guide actin remodeling. It is reported that light at different wavelengths and fluences can influence cytoskeleton behavior of varius cells. Therefore, priming SCs with PBM can have the following important implications: (i) better migration and proliferation post‐PBM, (ii) priming of SCs before implantation can lead to augmented migration in vivo. While PBM‐induced aspects such as enhanced proliferation and differentiation in hADMSCs have been documented [28], the efficacy of PBM to elicit improved cell migration has received little attention so far. Moreover, the PBM parameters like wavelength and the optimum fluence for eliciting cell migration are yet to be standardized. Therefore, our investigation adds new perspectives to PBM‐based SC stimulation.

### 3.1 Red Light (~660 nm) Exposure‐Induced Cellular ROS

**FIGURE 2:**
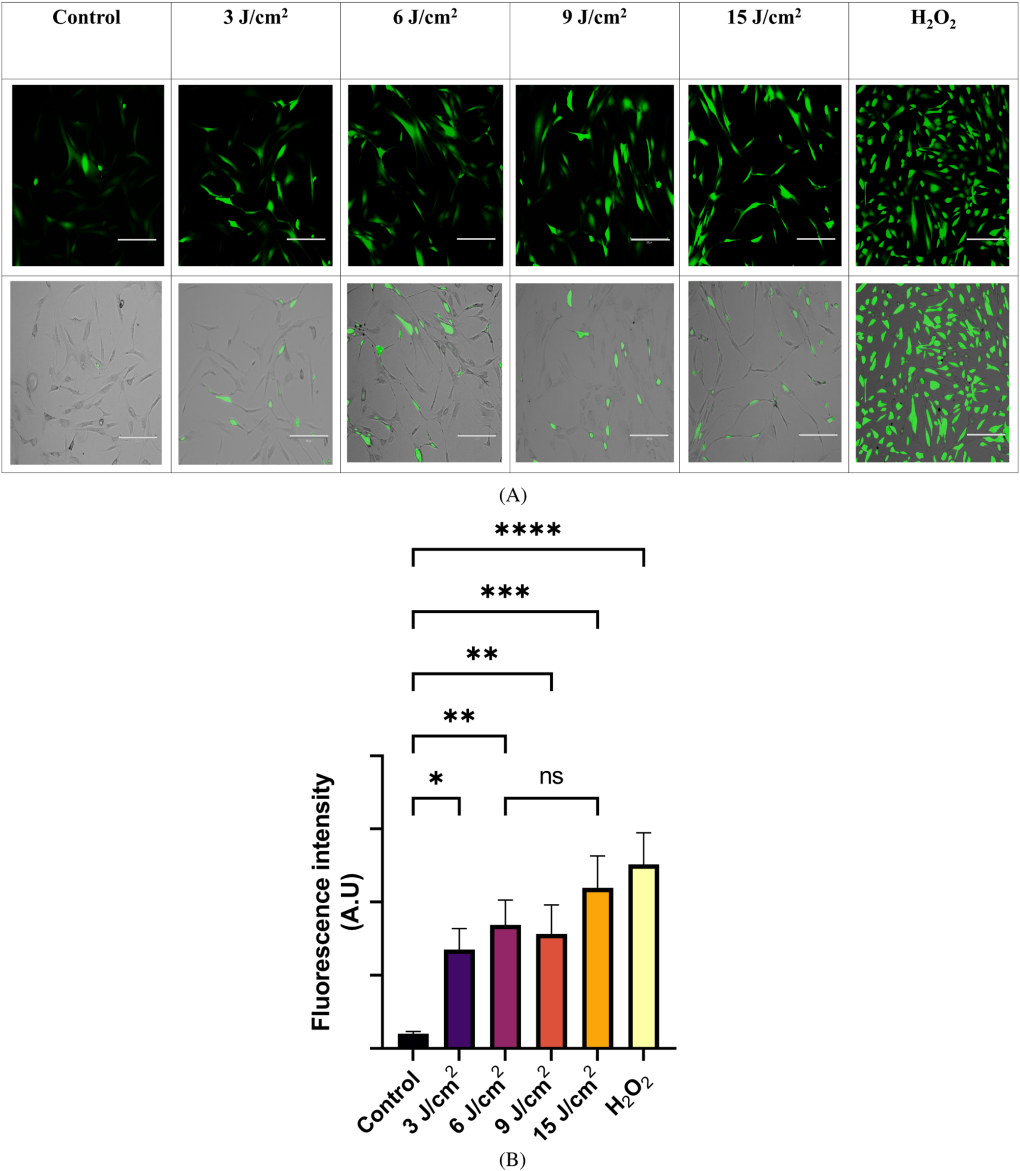
Red light (~660 nm) exposure‐induced cellular reactive oxygen species. (A) Fluorescence microscopic analysis of cellular ROS generation post red light (~660 nm) exposure. Scale bar = 100 μm. (B) Histogram showing analysis of ROS fluorescence intensity carried out using ImageJ. (ns = nonsignificant), *p ≤ 0.05, **p ≤ 0.01, ***p ≤ 0.001, and ****p ≤ 0.0001.

We first evaluated the increase in cellular ROS levels post‐PBM via fluorescence microscopy. Fluorescence microscopic data suggest PBM significantly increases the cellular ROS levels (Figure 2A). Our data reveal saturation of ROS levels at ~6 J/cm^2^ red light (~660 nm) fluence exposure, as we did not observe a proportionate increase in ROS levels with an increase in light fluence up to ~15 J/cm^2^ (Figure 2B).

### 3.2 Red Light (~660 nm) Exposure‐Induced Changes in F‐Actin Organization of hADMSCs

**FIGURE 3:**
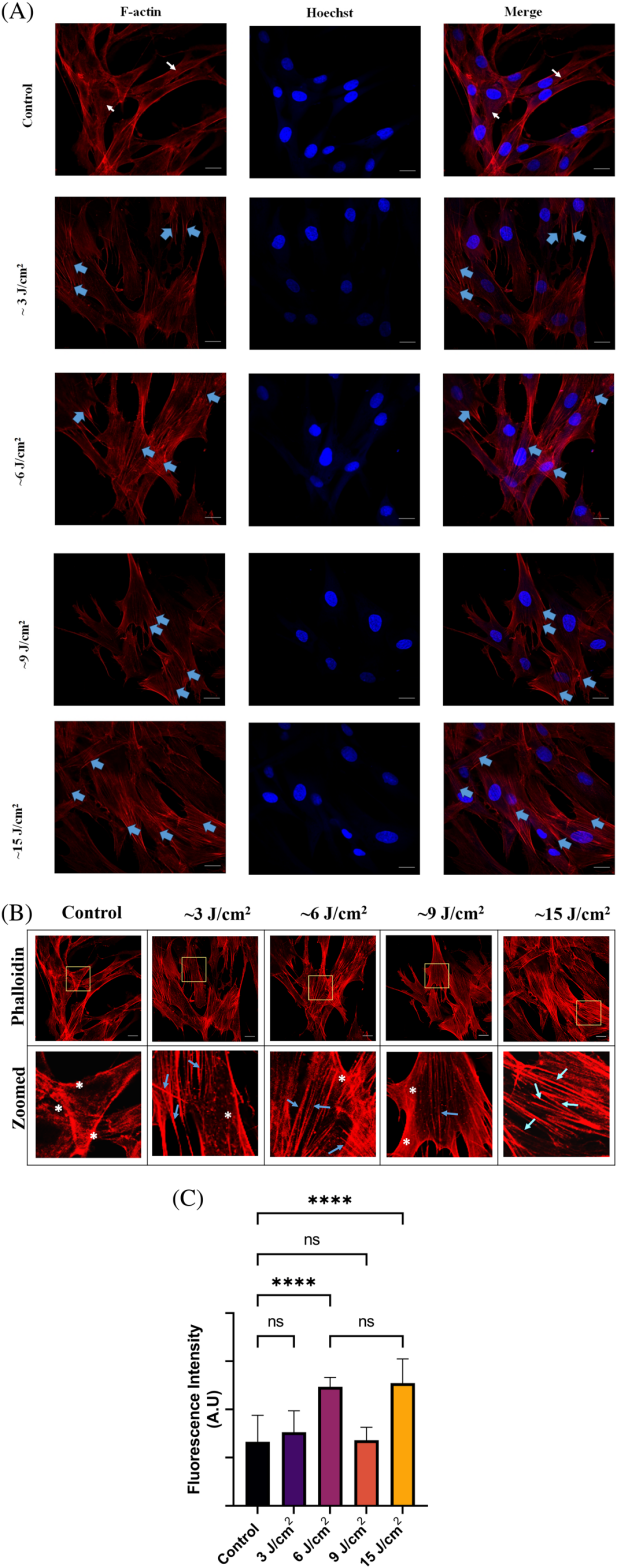
Red light (~660 nm) exposure‐induced changes in F‐actin organization. (A) Confocal analysis of F‐actin cytoskeleton performed immediately after cell exposure to red light. Scale bar = 20 μm. (B) Actin filament densification (scale bar = 10 μm), images in the bottom row represents magnification of the boxed area in the upper row (scale bar = 2 μm). (C) Histogram showing densitometric analysis of F‐actin fluorescence intensity carried out using ImageJ. ****p < 0.0001 (one‐way ANOVA).

Confocal images illustrate the modulation of F‐actin filaments stained with phalloidin. The densification of F‐actin filaments throughout the cell likely resulted from red light irradiation. In addition, the number of F‐actin filaments in the cytosol appeared to increase posttreatment, possibly due to the spreading of filaments in all directions (Figure 3A–C). This means that the actin filaments are very responsive to the action of external stimuli, such as PBM treatment, which has been used in this work. The density of F‐actin filaments increased in all the light‐exposed groups. This increase in F‐actin filaments appears to correlate with light fluence. This is evident in the confocal images, wherein, the ~15 J/cm^2^ fluence group shows a greater F‐actin density compared to the control group. Exposure to very high fluence (~60 J/cm^2^) leads to complete disorganization of the cytoskeleton (Data S1).

It is known that actin filaments tend to change continuously at a rapid rate, and hence, the observation of differences in the actin filaments, immediately after PBM, is reasonable. Our data is in agreement with another study, which observed actin reorganization within 5 min, in 3T3 fibroblast cells exposed to 625 nm (~35 J/cm^2^) and 808 nm (~38 J/cm^2^) [24]. Another study showed that irradiation of bone marrow cells with 808 nm at a fluence of ~60 J/cm^2^ for 60 s resulted in the thickening of actin filaments, which were observed to run parallel to form an expanded membrane [23]. Our results are also in qualitative accordance with the results of this study. However, in our study, the actin cytoskeleton changes are observed at a much lower fluence of ~6–15 J/cm^2^.

### 3.3 Red Light (~660 nm) Exposure‐Induced Changes in Mitochondrial Morphology of hADMSCs

**FIGURE 4:**
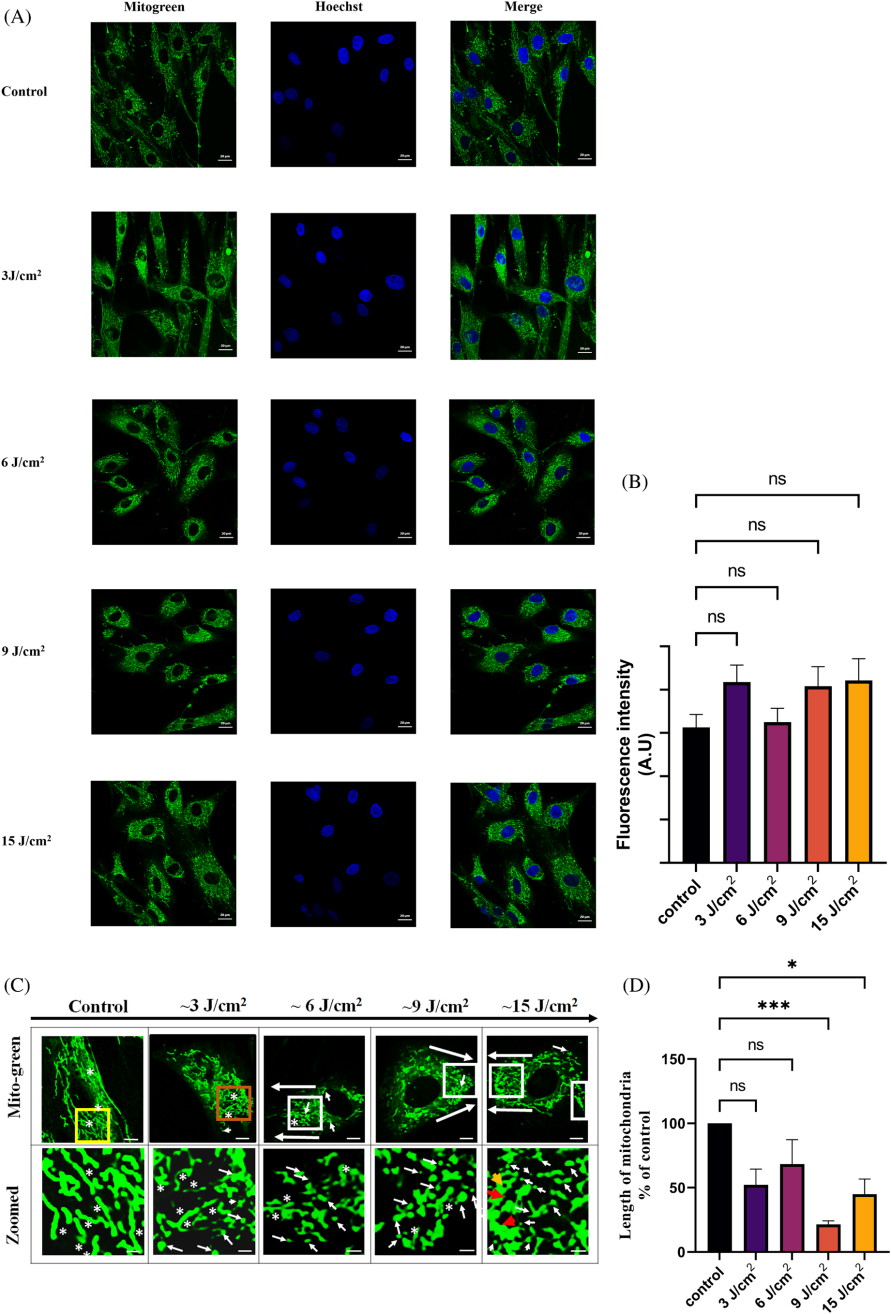
Red light (~660 nm) exposure‐induced changes in mitochondrial morphology. (A) Confocal analysis of mitochondrial biomass performed 2 h after cell exposure to red light (~660 nm). Scale bar = 20 μm. (B) Histogram showing analysis of mitochondrial biomass fluorescence intensity carried out using ImageJ. (ns = nonsignificant). (C) Mitochondrial morphological characteristics (scale bar = 10 μm). Images in the bottom row represent magnification of the boxed area in the upper row (scale bar = 2 μm). White squares in the images belonging to ~6, 9, and 15 J/cm^2^ exposure groups contain granular mitochondria (white arrowheads). The yellow squares in the unirradiated group contain more branched and elongated mitochondria (white stars). The orange square in the images belonging to 3 J/cm^2^ contains mixed; elongated (white stars) and granular (white arrow heads) type of mitochondria. (D) Mitochondrial length for each fluence group, *p < 0.05, ***p < 0.001 compared to control.

The results on mitochondrial morphology are represented in (Figure 4A–D), which show tubular, branched mitochondrial morphology in the unirradiated cells. The hADMSCs exposed to ~660 nm show granular mitochondria in the periphery, mainly in lamellipodia, invadopodia, and so forth, which represents the accumulation of dividing mitochondria at the migratory end of cells. Apart from the distribution, an important observation is that the mitochondria have undergone vivid morphological changes (Table 2) in a fluence‐dependent manner. The presence of branched mitochondria shows the decreasing trend, from the unirradiated group to 15 J/cm^2^ (Table 2). The fluorescence intensity in all the irradiated groups, except ~6 J/cm^2^, was slightly higher than that of the unirradiated group (Figure 4B). This increase is in accordance with the increased fluorescence observed for ischemic fibroblasts exposed to ~660 nm [29]. However, the total fluorescence increase is not significant (Figure 4B). At the same time, compared to cells of the untreated control, in the case of the irradiated cells, the length of mitochondria is considerably smaller (Figure 4C,D).

**TABLE 2:** Conspicuous features in actin cytoskeleton and mitochondrial morphology following ~660 nm light exposure.

Actin cytoskeleton	
Unirradiated	Diffused actin monomers Less number of actin fibers running parallel to the leading front of growth
Light irradiated	Increase in number of actin fibers running parallel to leading front of growth Presence of actin fibers: ~3 < ~6 < ~9 < ~15 J/cm^2^ Disorganization of actin fibers in very high fluence, ~60 J/cm^2^ (Supporting Information) Increase in fluorescence intensity
Mitochondrial morphology	
Unirradiated	Branched, elongated mitochondria Less number of granulated mitochondria Cytosolic distribution of mitochondria
Light irradiated	Smaller, granulated mitochondria Presence of granulated mitochondria: ~3 < ~6 < ~9 < ~15 J/cm^2^ Presence of chain like, elongated mitochondria in a cell: ~3 > ~6 > ~9 > ~15 J/cm^2^ Peripheral distribution of granulated mitochondria, near lamellipodia and invadopodia Ring like, circular mitochondria in high fluence group (~15 J/cm^2^) Slight increase in fluorescence intensity (20%–30%) The percent of cells under microscopic field of view showing granulated mitochondria: ~3 < ~6 < ~9 < ~15 J/cm^2^

### 3.4 Red Light (~660 nm) Exposure‐Induced Changes in hADMSCs Migration

**FIGURE 5:**
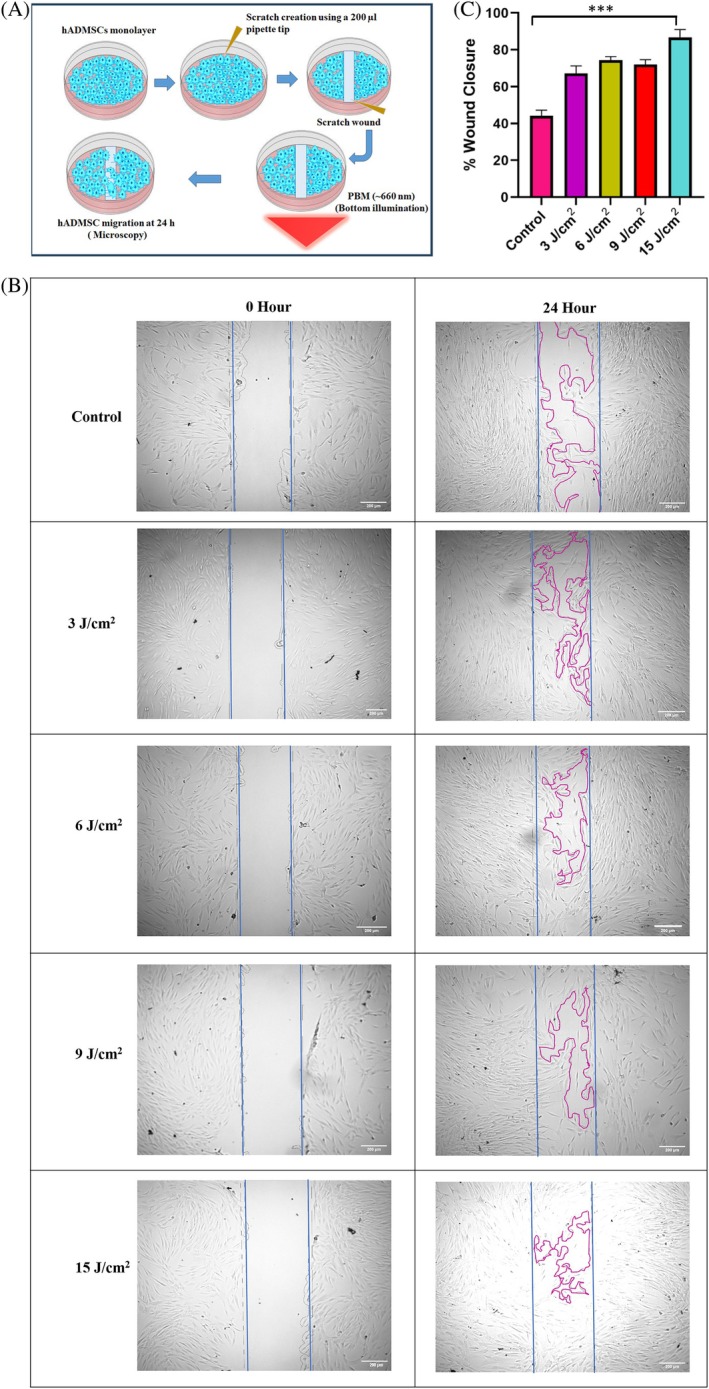
Red light (~660 nm) exposure‐induced changes in hADMSCs migration. (A) Experimental scheme of scratch assay. (B) Effect of different red light fluence on hADMSC cell migration at 24 h as measured by light microscopy (scale bar = 200 μm). (C) Compiled data of wound closure rate from three independent experiments. Columns, mean; bars, ±SD, ***p < 0.001.

The results of the scratch wound assay are presented in Figure 5B,C. These results show that the irradiated cells appeared normal in morphology (regular spindle‐shaped structure) with increased cell migration toward the central scratch over time. In the case of the ~15 J/cm^2^ group, complete wound closure occurred at 24 h. Unirradiated cells showed comparatively slower cell migration toward the central scratch, resulting in a delay in wound closure at 24 h (Figure 5B,C). The percent wound closure, quantified with ImageJ, at 24 h with respect to the initial time point in the control, ~3, 6, 9, and 15 J/cm^2^ red light exposure groups was approximately ~44%, ~67%, ~74%, ~72%, and ~86%, respectively.

### 3.5 Red Light (~660 nm) Exposure‐Induced Changes in ATP Levels

**FIGURE 6:**
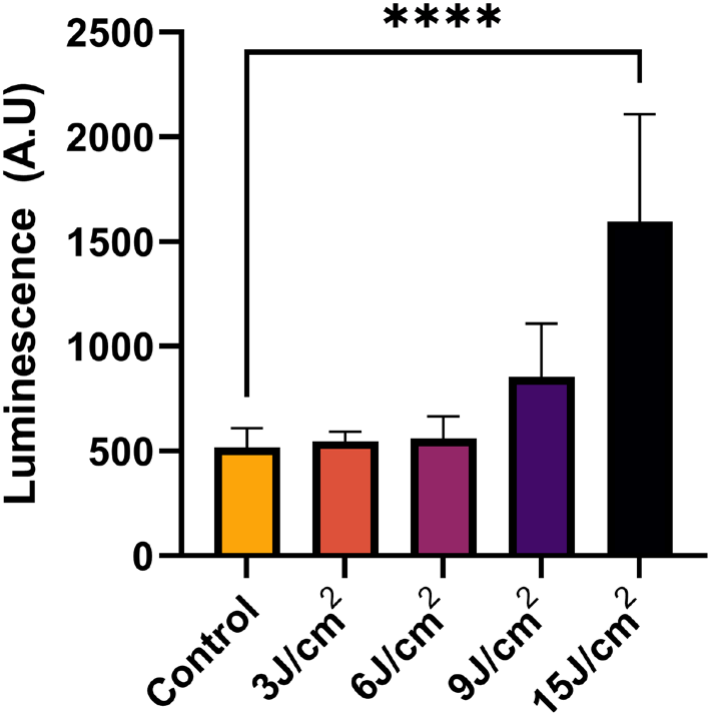
Red light (~660 nm) exposure‐induced changes in ATP levels in hADMSCs immediately post ~660 nm light exposure. Data represent mean ± SD, *p < 0.05, ****p < 0.0001.

To evaluate the effect of red light exposure on cellular ATP levels, we measured the ATP levels in hADMSCs post‐PBM. Our results show that, as compared to unirradiated cells, red light exposure at ~15 J/cm^2^ enhances (2.5–3 times) ATP levels significantly (Figure 6).

### 3.6 Red Light (~660 nm) Exposure‐Induced Changes in hADMSCs Cell Viability and Proliferation

**FIGURE 7:**
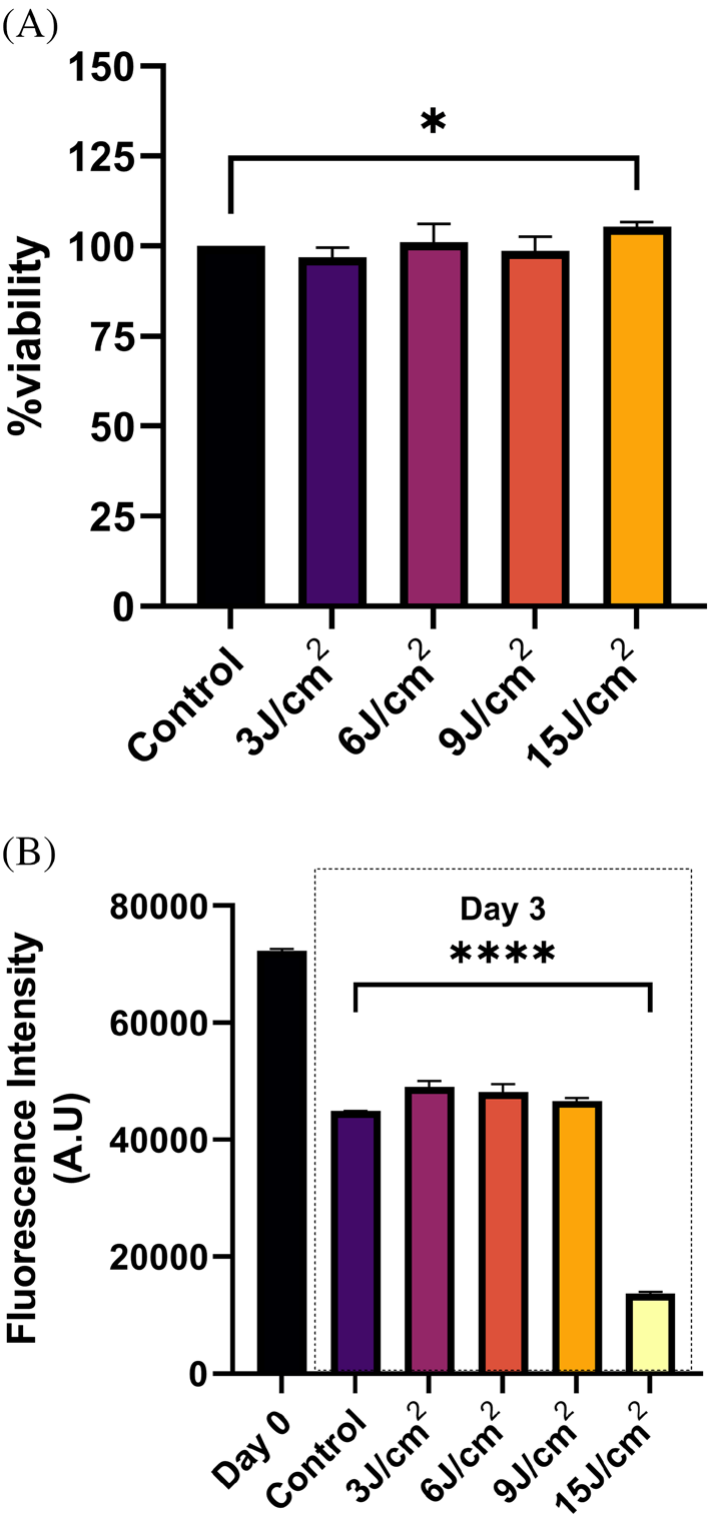
Red light (~660 nm) exposure‐induced changes in hADMSCs cell viability and proliferation. (A) Effect of red light (~660 nm) exposure on cell viability at 24 h, as measured by WST assay. *p < 0.05 (Student *t*‐test). (B) Effect of red light (~660 nm) exposure on cell proliferation at 72 h, as measured by CFSE assay. ****p ≤ 0.0001 (one‐way ANOVA).

To determine if 660 nm based PBM can lead to any change in cell viability and cell proliferation, we performed WST‐8 and CFSE cell proliferation assays, respectively. Results of the WST‐8 assay (Figure 7A) showed that at 24 h post ~660 nm light exposure, the group irradiated with ~15 J/cm^2^ presented higher cell viability in comparison to other irradiated and unirradiated groups. However, we did not observe a significant difference among the remaining light‐irradiated groups. The results of the CFSE cell proliferation assay (Figure 7B) at 72 h after irradiation indicate that red light exposure at ~15 J/cm^2^ leads to stimulation of stem cell proliferation. Likewise, in the CFSE cell proliferation assay, we also did not find any significant difference among the remaining groups.

Although mitochondrial fission and fusion dynamics can be controlled by various internal and external stimuli, ROS has been observed to be one of the common denominators. It has been demonstrated that ROS can regulate cytoskeletal and mitochondrial remodeling to harmonize cell and tissue dynamics [30]. Mitochondria can accomplish the intensive energy demand required for wound repair and closure by cytoskeleton remodeling via morphological alteration and mitochondrial reactive oxygen species (mtROS) signaling [31]. In this context, PBM using ~660 nm is known to generate low‐level ROS, and our results (Figure 2) also showed enhanced ROS levels following ~660 nm PBM. Therefore, the mitochondrial granulation observed in our study can be partly attributed to ~660 nm light exposure‐induced ROS generation. The morphological changes in mitochondria have been observed to be intimately linked to overall mitochondrial activity, influencing the production of both ATP and mtROS, which allow mitochondria to respond to external stimuli and adapt to various cellular demands [32]. Low amounts of mtROS that trigger granular mitochondria formation are distinct from fragmented mitochondria observed in high ROS‐induced cell death, apoptotic cells [33]. The mitochondrial fission cycles, which occur continuously, are increasingly being implicated to affect both mitochondrial and cell phenotypes important for the progression to migratory phenotype [34], for which the fragmented pools of mitochondria tend to gather at and influence the formation of membrane protrusions, including lamellipodia and invadopodia [34]. This granulation of mitochondria ultimately drives enhancement in cell motility and invasion [34]. Previously, it has been reported that ~630 nm exposed Hep‐2 cells showed mitochondrial granular appearance after 1 h of light exposure [35]. However, the relevance and mechanism of such an observation at the cellular level were not clear from their study. Moreover, Hep and SCs have completely different properties, mitochondrial pools, and migration behavior. Therefore, our study is novel in respect that it is the first study to report ~660 nm PBM‐induced localization of granular mitochondria in SCs, which can be related to cell migration.

It can be discerned from the results in Figure 5 that the magnitude of increase in cell migration is more pronounced at a fluence range of ~15 J/cm^2^. The difference in the magnitude of changes observed for the scratch wound and CFSE dilution‐based cell proliferation assay (Figure 7B) are expected, but, within the fluence range reported in previous studies on PBM involving ~660 nm light exposure [19]. The higher magnitude of changes observed in the scratch wound versus MTT assay might be due to the different assay protocols; it can also be ascribed to the fact that injured or damaged/stressed cells tend to respond to PBM much better compared to healthy and unstressed counterparts. In the MTT/WST assay, while mitochondrial dehydrogenase activity is monitored in healthy cells, the scratch wound assay determines the migration potential of damaged and stressed cells near the scratch/wounded region. The increased cell migration (Figure 5A–C) and proliferation (Figure 7B) can also be ascribed to cytoskeleton reinforcement and mitochondrial division, verified by confocal microscopy and increased ATP production (Figure 6). Whether the PBM‐induced actin remodeling governs mitochondrial division requires further detailed investigations at the transcriptional and translational levels.

The induction of cytoskeleton remodeling by PBM has been demonstrated in some studies on non‐stem cells. Recently, in a study on 3T3 mouse fibroblasts, it was reported that PBM can alter the cytoskeleton [24]. Mokoena et al. showed increased α‐smooth muscle actin in an injured model of fibroblasts after stimulation by 660 nm (fluence ~5 J/cm^2^) based PBM [36]. Another recent study showed that 660 nm at fluence ~6 J/cm^2^ exposure resulted in improved migration of astrocytes [19]. However, there was no such systematic mechanistic study on red light PBM‐induced cell migration in any kind of SCs, which possess different metabolism, migration properties, and bioenergetics than other primary cells. Thus, our study not only adds valuable understanding of ~660 nm PBM‐induced F‐actin alteration and cell motility in any kind of SCs, but also indicates another way of modulating SC function in vitro. As red light is the most studied wavelength in PBM and the red‐light‐activable mitochondrial chromophores, such as CCO, are constitutively expressed in cells, including SCs, a further clinical translation utilizing 660 nm PBM‐based preconditioning of SCs would be easier.

**FIGURE 8:**
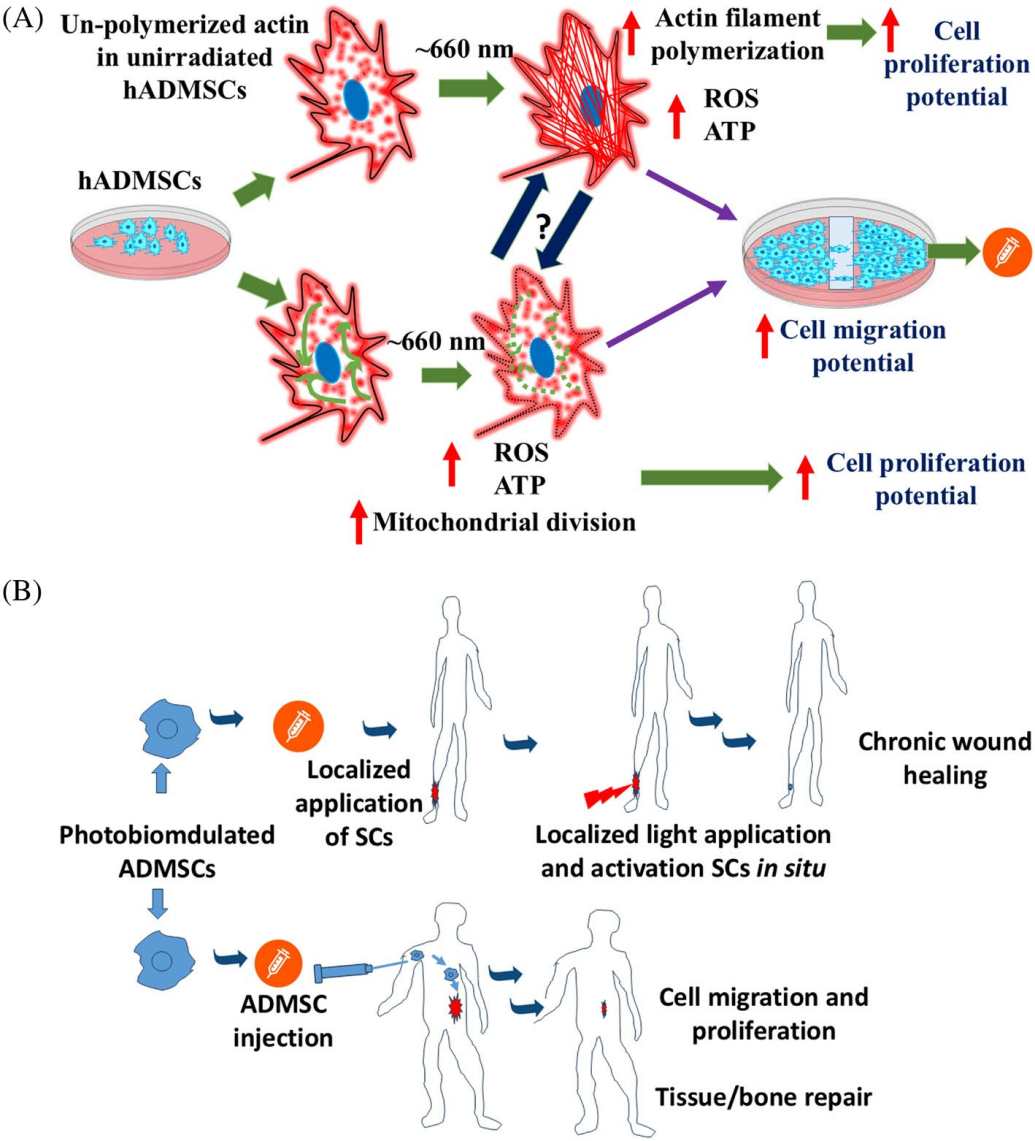
(A) Putative mechanism of ~660 nm action on hADMSC cytoskeleton and mitochondrial state modulation. (B) Possibility of PBM‐based priming of hADMSC preimplantation and postimplantation for therapeutic applications.

Very recently, by employing micro‐Raman (micro‐RS) spectroscopy, we demonstrated that 660 nm light leads to the activation of reduced cytochrome c and CCO [37]. It is noteworthy that CCO activity is linked to actin ROS production and mitochondrial dynamics as well as ATP generation [38]. Even though we have not measured mitochondrial membrane potential in this study, our results of the current study on ROS (Figure 2), ATP generation (Figure 6), mitochondrial morphological changes (Figure 4), and previous micro‐RS results on CCO involvement as well as other studies reported elsewhere [37] indicate the involvement of mitochondrial activation, underneath the structural observations such as actin filament rearrangement and mitochondrial morphology. Our data, compared to the previously published data [18], add a new repertoire of information on cell migration alteration. Therefore, based on our recently published data and the results of the current study on increased ROS and ATP (Figures 2 and 6), a putative mechanism of ~660 nm action on hADMSCs cytoskeleton densification, rearrangement, and mitochondrial morphological dynamic alteration based on improved cell migration and proliferation can be proposed (Figure 8). The chromophore excitation is followed by an increase in ROS and ATP, leading to rapid modulation of the actin cytoskeleton in minutes, which in turn can contribute to cell migration directly or indirectly via guiding mitochondrial division (Figure 8A). In the future, it would be prudent to decipher if there is any intersection in these direct or indirect pathways. Furthermore, the complete destabilization of the actin filament at ~60 J/cm^2^ (Data S1) again reiterates the need to optimize the fluence to a range so as to achieve the best cell migration outcome. Recently, a cochlear stem cell implantation model‐based study showed the possibility of stem cell manipulation in vivo by PBM [39]. Therein, PBM treatment (808 nm, 40 mW/cm^2^ for 1 h/day, 5 days) could enhance SC viability in the cochlea with auditory neuropathy. The possibility that ~660 nm exposure can enhance cellular migration, a controlled pre‐priming of the hADMSCs pre‐ and post‐implantation using PBM can be a very interesting, cost‐effective prospect for improving SC homing in localized and topical applications such as wound healing (Figure 8B). However, the irradiation conditions in vivo present significant challenges, so a direct comparison between in vitro results and in vivo outcomes may not be appropriate. Therefore, more studies on suitable preclinical and clinical studies will fully validate these conjectures. Furthermore, a detailed and systematic study on the activation of genes and signaling pathways responsible for mitochondrial activation, biogenesis, correlating with actin polymerization, and MMP alterations following 660 nm light exposure is warranted.

## 4 Conclusion

In conclusion, the effect of ~660 nm exposure on the actin cytoskeleton, mitochondrial morphological rearrangement, and migration of hADMSC was studied. Furthermore, the PBM effect on ROS, ATP production, cell viability, and cell proliferation was investigated. Our results indicate that PBM at ~660 nm could modulate changes in actin cytoskeleton organization and mitochondrial morphology. These ultrastructural changes were observed in conjunction with increased cell migration in the scratch wound, enhancement in ROS, ATP levels, and the reinforcement of SC function. However, a suitable preclinical model and clinical studies will fully validate the exact in vivo potential of these observations.

## Conflicts of Interest

The authors declare no conflicts of interest.

## Supporting information


**Data S1:** Red light (~660 nm) high fluence (~60 J/cm^2^) exposure induced actin filament disorganization in vitro. Confocal microscopic images of hADMSCs. Left panel: rhodamine phalloidin stained image. Central Panel: Hoechstt 33342 stained image. Right panel: Merged fluorescent image. Blue arrows: Disorganized actin filaments.

## Data Availability

The data that support the findings of this study are available on request from the corresponding author. The data are not publicly available due to privacy or ethical restrictions.

## References

[1] Y. Jin , S. Li , Q. Yu , T. Chen , and D. Liu , “Application of Stem Cells in Regeneration Medicine,” MedComm 4, no. 4 (2023): e291, 10.1002/mco2.291. 37337579 PMC10276889

[2] D. S. Chulpanova , K. V. Kitaeva , L. G. Tazetdinova , V. James , A. A. Rizvanov , and V. V. Solovyeva , “Application of Mesenchymal Stem Cells for Therapeutic Agent Delivery in Anti‐Tumor Treatment,” Frontiers in Pharmacology 9 (2018): 259, 10.3389/fphar.2018.00259. 29615915 PMC5869248

[3] S. Dekoninck and C. Blanpain , “Stem Cell Dynamics, Migration and Plasticity During Wound Healing,” Nature Cell Biology 21, no. 1 (2019): 18–24, 10.1038/s41556-018-0237-6. 30602767 PMC7615151

[4] B. de Lucas , L. M. Pérez , and B. G. Gálvez , “Importance and Regulation of Adult Stem Cell Migration,” Journal of Cellular and Molecular Medicine 22, no. 2 (2018): 746–754, 10.1111/jcmm.13422. 29214727 PMC5783855

[5] S. H. Hong , M. H. Lee , M. Koo , Y. J. Park , and D. Kim , “Stem Cell Passage Affects Directional Migration of Stem Cells in Electrotaxis,” Stem Cell Research 38 (2019): 101475, 10.1016/j.scr.2019.101475. 31176110

[6] M. A. Kinney and T. C. McDevitt , “Emerging Strategies for Spatiotemporal Control of Stem Cell Fate and Morphogenesis,” Trends in Biotechnology 31, no. 2 (2013): 78–84, 10.1016/j.tibtech.2012.11.001. 23219200 PMC3557560

[7] M. C. P. Felician , R. Belotto , J. P. Tardivo , M. S. Baptista , and W. K. Martins , “Photobiomodulation: Cellular, Molecular, and Clinical Aspects,” Journal of Photochemistry and Photobiology 17 (2023): 100197, 10.1016/j.jpap.2023.100197.

[8] T. Kushibiki , T. Hirasawa , S. Okawa , and M. Ishihara , “Low Reactive Level Laser Therapy for Mesenchymal Stromal Cells Therapies,” Stem Cells International 2015 (2015): 974864, 10.1155/2015/974864. 26273309 PMC4529981

[9] E. Balta , J. Kramer , and Y. Samstag , “Redox Regulation of the Actin Cytoskeleton in Cell Migration and Adhesion: On the Way to a Spatiotemporal View,” Frontiers in Cell and Developmental Biology 8 (2021): 618261, 10.3389/fcell.2020.618261. 33585453 PMC7875868

[10] V. B. Dugina , G. S. Shagieva , A. S. Shakhov , and I. B. Alieva , “The Cytoplasmic Actins in the Regulation of Endothelial Cell Function,” International Journal of Molecular Sciences 22, no. 15 (2021): 7836, 10.3390/ijms22157836. 34360602 PMC8345992

[11] M. Illescas , A. Peñas , J. Arenas , M. A. Martín , and C. Ugalde , “Regulation of Mitochondrial Function by the Actin Cytoskeleton,” Frontiers in Cell and Developmental Biology 9 (2021): 795838, 10.3389/fcell.2021.795838. 34993202 PMC8725978

[12] X. Xie , T. Venit , N. Drou , and P. Percipalle , “In Mitochondria β‐Actin Regulates mtDNA Transcription and Is Required for Mitochondrial Quality Control,” iScience 3 (2018): 226–237, 10.1016/j.isci.2018.04.021. 30428323 PMC6137402

[13] M. Shah , L. A. Chacko , J. P. Joseph , and V. Ananthanarayanan , “Mitochondrial Dynamics, Positioning and Function Mediated by Cytoskeletal Interactions,” Cellular and Molecular Life Sciences 78, no. 8 (2021): 3969–3986, 10.1007/s00018-021-03762-5. 33576841 PMC11071877

[14] S. Varland , J. Vandekerckhove , and A. Drazic , “Actin Post‐Translational Modifications: The Cinderella of Cytoskeletal Control,” Trends in Biochemical Sciences 44, no. 6 (2019): 502–516, 10.1016/j.tibs.2018.11.010. 30611609

[15] J.‐S. Kim , T. Y. Huang , and G. M. Bokoch , “Reactive Oxygen Species Regulate a Slingshot‐Cofilin Activation Pathway,” Molecular Biology of the Cell 20 (2009): 2650–2660, 10.1091/mbc.e09-02-0131. 19339277 PMC2688545

[16] C. Cinq‐Frais , C. Coatrieux , A. Savary , et al., “Annexin II‐Dependent Actin Remodelling Evoked by Hydrogen Peroxide Requires the Metalloproteinase/Sphingolipid Pathway,” Redox Biology 4 (2015): 169–179, 10.1016/j.redox.2014.12.005. 25574848 PMC4309845

[17] T. Ishimoto and H. Mori , “Control of Actin Polymerization via Reactive Oxygen Species Generation Using Light or Radiation,” Frontiers in Cell and Development Biology 10 (2022): 1014008, 10.3389/fcell.2022.1014008. PMC9538341 36211457

[18] A. B. Shoorche , A. Mohammadkarim , M. Jadidi , and M. Bahraminasab , “Photobiomodulation Therapy Affects the Elastic Modulus, Cytoskeletal Rearrangement and Migration Capability of Human Osteosarcoma Cells,” Lasers in Medical Science 37, no. 7 (2022): 2855–2863, 10.1007/s10103-022-03554-8. 35394552

[19] S. R. Yoon , S.‐Y. Chang , M. Y. Lee , and J.‐C. Ahn , “Effects of 660‐nm LED Photobiomodulation on Drebrin Expression Pattern and Astrocyte Migration,” Scientific Reports 13 (2023): 6220, 10.1038/s41598-023-33469-5. 37069238 PMC10110518

[20] Y. Wang , Y. Y. Huang , Y. Wang , P. Lyu , and M. R. Hamblin , “Red (660 nm) or Near‐Infrared (810 nm) Photobiomodulation Stimulates, While Blue (415 nm), Green (540 nm) Light Inhibits Proliferation in Human Adipose‐Derived Stem Cells,” Scientific Reports 7 (2017): 7781, 10.1038/s41598-017-07525-w. 28798481 PMC5552860

[21] F. Zare , A. Moradi , S. Fallahnezhad , et al., “Photobiomodulation With 630 Plus 810 nm Wavelengths Induce More In Vitro Cell Viability of Human Adipose Stem Cells Than Human Bone Marrow‐Derived Stem Cells,” Journal of Photochemistry and Photobiology B: Biology 201 (2019): 111658, 10.1016/j.jphotobiol.2019.111658. 31710923

[22] E. Malthiery , B. Chouaib , A. M. Hernandez‐Lopez , et al., “Effects of Green Light Photobiomodulation on Dental Pulp Stem Cells: Enhanced Proliferation and Improved Wound Healing by Cytoskeleton Reorganization and Cell Softening,” Lasers in Medical Science 36, no. 2 (2021): 437–445, 10.1007/s10103-020-03092-1. 32621128

[23] A. Amaroli , M. G. Sabbieti , L. Marchetti , et al., “The Effects of 808‐nm Near‐Infrared Laser Light Irradiation on Actin Cytoskeleton Reorganization in Bone Marrow Mesenchymal Stem Cells,” Cell and Tissue Research 383 (2021): 1003–1016, 10.1007/s00441-020-03306-6. 33159579

[24] A. C. de Magalhães , Z. Guimarães‐Filho , E. M. Yoshimura , and L. Lilge , “Photobiomodulation Therapy Can Change Actin Filaments of 3T3 Mouse Fibroblast,” Lasers in Medical Science 35 (2020): 585–597, 10.1007/s10103-019-02852-y. 31410615

[25] G. Benard and M. Karbowski , “Mitochondrial Fusion and Division: Regulation and Role in Cell Viability,” Seminars in Cell & Developmental Biology 20, no. 3 (2009): 365–374, 10.1016/j.semcdb.2008.12.012. 19530306 PMC2768568

[26] T. F. Alvear , A. Farias‐Pasten , S. A. Vergara , et al., “Hemichannels Contribute to Mitochondrial Ca^2+^ and Morphology Alterations Evoked by Ethanol in Astrocytes,” Frontiers in Cell and Developmental Biology 12 (2024): 1434381, 10.3389/fcell.2024.1434381. 39129788 PMC11310047

[27] G. Sharapova , G. Sharapova , G. Sharapova , et al., “Mitochondrial Protein Density, Biomass, and Bioenergetics as Predictors for the Efficacy of Glioma Treatments,” International Journal of Molecular Sciences 25, no. 13 (2024): 7038, 10.3390/ijms25137038. 39000148 PMC11241254

[28] A. Etemadi , M. Aghaie , F. Sayar , and N. Chiniforush , “Effect of Photobiomodulation Therapy With 660 and 980 nm Diode Lasers on Differentiation of Periodontal Ligament Mesenchymal Stem Cells,” Scientific Reports 14 (2024): 20587, 10.1038/s41598-024-71386-3. 39232133 PMC11375153

[29] N. N. Houreld , R. T. Masha , and H. Abrahamse , “Low‐Intensity Laser Irradiation at 660 nm Stimulates Cytochrome c Oxidase in Stressed Fibroblast Cells,” Lasers in Surgery and Medicine 44, no. 5 (2012): 429–434, 10.1002/lsm.22027. 22488690

[30] S. Muliyil and M. Narasimha , “Mitochondrial ROS Regulates Cytoskeletal and Mitochondrial Remodeling to Tune Cell and Tissue Dynamics in a Model for Wound Healing,” Developmental Cell 28, no. 3 (2014): 239–252, 10.1016/j.devcel.2013.12.019. 24486154

[31] S. Xu , S. Li , M. Bjorklund , and S. Xu , “Mitochondrial Fragmentation and ROS Signaling in Wound Response and Repair,” Cell Regeneration 11 (2022): 38, 10.1186/s13619-022-00141-8. 36451031 PMC9712903

[32] R. Sabouny and T. E. Shutt , “Reciprocal Regulation of Mitochondrial Fission and Fusion,” Trends in Biochemical Sciences 45, no. 7 (2020): 564–577, 10.1016/j.tibs.2020.03.009. 32291139

[33] C. Cid‐Castro and J. Morán , “Differential ROS‐Mediated Phosphorylation of Drp1 in Mitochondrial Fragmentation Induced by Distinct Cell Death Conditions in Cerebellar Granule Neurons,” Oxidative Medicine and Cellular Longevity 2021 (2021): 8832863, 10.1155/2021/8832863. 33936388 PMC8060094

[34] D. P. Boulton and M. C. Caino , “Mitochondrial Fission and Fusion in Tumor Progression to Metastasis,” Frontiers in Cell and Development Biology 10 (2022): 849962, 10.3389/fcell.2022.849962. PMC8959575 35356277

[35] R. Bortoletto , N. S. Silva , R. A. Zângaro , M. T. T. Pacheco , R. A. da Matta , and C. Pacheco‐Soares , “Mitochondrial Membrane Potential After Low‐Power Laser Irradiation,” Lasers in Medical Science 18, no. 4 (2004): 204–206, 10.1007/s10103-003-0281-7. 15042424

[36] D. R. Mokoena , N. N. Houreld , S. S. Dhilip Kumar , and H. Abrahamse , “Photobiomodulation at 660 nm Stimulates Fibroblast Differentiation,” Lasers in Surgery and Medicine 52, no. 7 (2020): 671–681, 10.1002/lsm.23204. 31820475

[37] M. S. Rastogi , A. Chowdhury , S. Chakraborty , K. Sahu , and S. K. Majumder , “Label‐Free and Real‐Time Assessment of 660 nm Red Light Photobiomodulation Induced Molecular Alterations in Human Adipose Derived Mesenchymal Stem Cells Using Micro Raman Spectroscopy,” Spectrochimica Acta Part A: Molecular and Biomolecular Spectroscopy 329 (2025): 125552. 39647267 10.1016/j.saa.2024.125552

[38] L.‐C. Pan , N.‐L.‐T. Hang , M. M. S. Colley , et al., “Single Cell Effects of Photobiomodulation on Mitochondrial Membrane Potential and Reactive Oxygen Species Production in Human Adipose Mesenchymal Stem Cells,” Cells 11, no. 6 (2022): 972, 10.3390/cells11060972. 35326423 PMC8946980

[39] S. Y. Chang , E. Kim , N. T. Carpena , J.‐H. Lee , D. H. Kim , and M. Y. Lee , “Photobiomodulation Can Enhance Stem Cell Viability in Cochlea With Auditory Neuropathy but Does Not Restore Hearing,” Stem Cells International 2023 (2023): 6845571, 10.1155/2023/6845571. 38020205 PMC10665102

