# Supplementary figures and images for: Six Hundred and Sixty Nanometer Light Exposure‐Induced Alterations in Actin Filament, Mitochondrial Morphological Dynamics, and Migration in Mesenchymal Stem Cells
Source: J Biophotonics. 2025 Sep 5;19(1):e70137. doi: 10.1002/jbio.202400544 (PMC12809618; doi:10.1002/jbio.202400544)

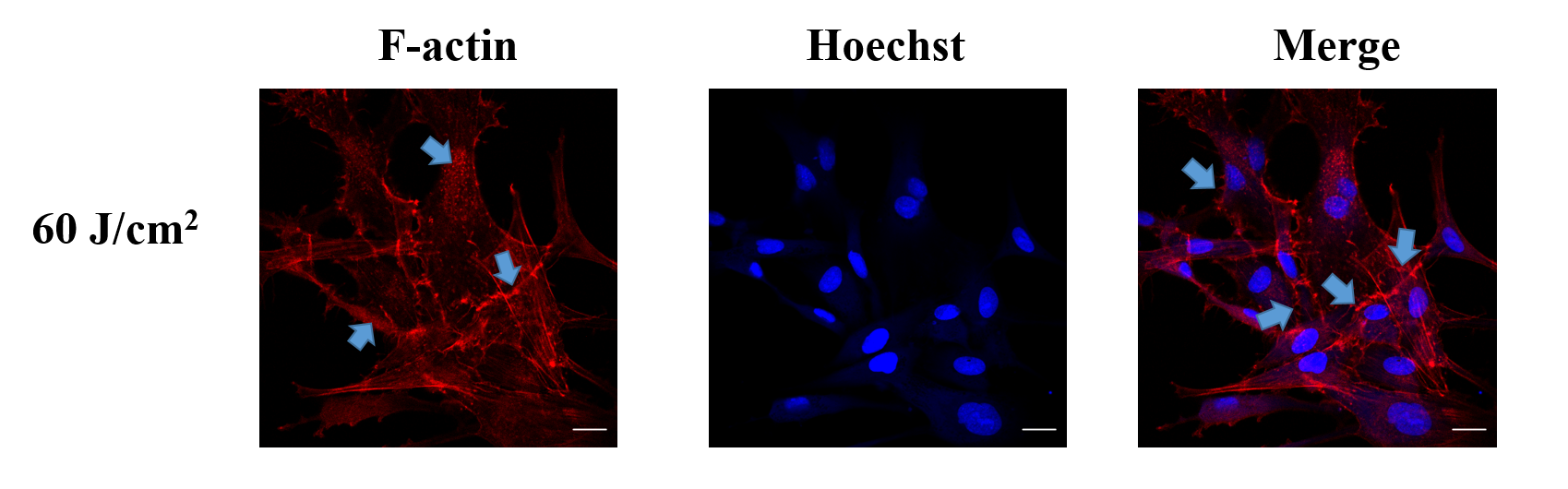

Supplement: Supplementary file 1 — Data S1: Red light (~660 nm) high fluence (~60 J/cm2) exposure induced actin filament disorganization in vitro. Confocal microscopic images of hADMSCs. Left panel: rhodamine phalloidin stained image. Central Panel: Hoechstt 33342 stained image. Right panel: Merged fluorescent image. Blue arrows: Disorganized actin filaments. [file JBIO-19-e70137-s001.tif]
